# PD98059 protects SH-SY5Y cells against oxidative stress in oxygen–glucose deprivation/reperfusion

**DOI:** 10.1515/tnsci-2022-0300

**Published:** 2023-09-06

**Authors:** Xiang-Zhen Zhuge, Wan-Xiang Hu, Yu-Mei Liu, Chang-Yue Jiang, Xiao-Hua Zhang, Meng-Hua Chen, Lu Xie

**Affiliations:** Department of Physiology, Pre-Clinical Science, Guangxi Medical University, 22 Shuangyong Road, Nanning, 350001, Guangxi, China; Shenzhen Bay Laboratory Neuropathy Institute of China, Shenzhen, 518107, Guangdong, China; Department of Pharmacy, HIV/AIDS Clinical Treatment Center of Guangxi (Nanning) and The Fourth People’s Hospital of Nanning, Nanning, 530000, China; Institute of Cardiovascular Diseases, The Second Hospital Affiliated to Guangxi Medical University, Nanning, 530000, Guangxi, China

**Keywords:** extracellular signal-regulated kinase, oxidative stress, mitochondria, apoptosis

## Abstract

Mitochondria play a key role in the cerebral ischemia-reperfusion injury. Although the extracellular signal-regulated kinase 1/2 inhibitor PD98059 (PD) is a selective and reversible flavonoid that can protect the mitochondria in a rat model of cardiac arrest/cardiopulmonary resuscitation, its role requires further confirmation. In this study, we investigated whether PD could maintain mitochondrial homeostasis and decrease reactive oxygen species (ROS) production in neuroblastoma (SH-SY5Y) cells exposed to oxygen–glucose deprivation/reperfusion (OGD/R). PD improved the mitochondrial morphology and function, reversed the increase in ROS production and cell apoptosis, and reduced total-superoxide dismutase and Mn-superoxide dismutase activities induced by OGD/R. PD decreases ROS production and improves mitochondrial morphology and function, protecting SH-SY5Y cells against OGD/R-induced injury.

## 1 Introduction

Cardiac arrest (CA) is a global health problem, accounting for 20% of all deaths in Western countries [1]. Brain injury after cardiopulmonary resuscitation (CPR) eventually leads to death [2]. A series of complex pathophysiological processes, including disordered energy metabolism, oxidative stress, mitochondrial injury, inflammation, and calcium overload, are involved in cerebral ischemia-reperfusion injury (CIRI) [3,4,5]. Studies on the mechanisms underlying CIRI will help improve prognosis and reduce mortality. Extracellular-regulated protein kinase (ERK) is activated by oxidative stress [6] and ischemia [7]. One study found that chromium caused an increase in reactive oxygen species (ROS) generation, ERK activation, and mitochondrial damage in SH-SY5Y cells, and PD improved its oxidative stress and mitochondrial dysfunction [8]. Dexmedetomidine and resveratrol have been shown to inhibit transient focal CIRI in rats [7,9]. Previously, we showed that PD98059 (PD), a specific ERK inhibitor, ameliorated oxidative stress and mitochondrial injury-related apoptosis and autophagy in a rat model of CA/CPR, conferring cerebral and renal protection [10,11,12]. Furthermore, antioxidative tea polyphenols and epigallocatechin gallate have been shown to alleviate CIRI in a dose-dependent manner via inhibition of ERK and modulation of mitochondrial function [13,14]. In an *in vitro* study, PAQR3 abated oxygen–glucose deprivation/reperfusion (OGD/R)-induced injury by suppressing ERK signaling in N2a cells [15]. Based on these findings, here, we established an SH-SY5Y model of OGD/R and evaluated mitochondrial morphology and function, oxidative stress, and cell apoptosis to further reveal the protective effects of PD.

## 2 Materials and methods

### 2.1 Cell culture and treatment groups

Human neuroblastoma SH-SY5Y cells were obtained from the China Center for Type Culture Collection (Wuhan, China). They were verified to be of the correct lineage and uncontaminated by other cell types or organisms. The cells were cultured in a mixed substrate, comprising a 1:1 mixture of Minimal Essential Medium (MEM) and F12 (Gibco BRL, Gaithersburg, MD, USA) and 10% fetal bovine serum (Gibco BRL) at 37°C under 5% CO_2_.

SH-SY5Y cells were stochastically separated into five groups as follows: normal control group (control), DMSO (solvent for PD) control group (DMSO), OGD/R group, PD low-dose group (PD 10 μmol/L, PD-L), and PD high-dose group (PD 20 μmol/L, PD-L).

### 2.2 OGD/R model

The OGD/R model was established as previously described [16]. For the OGD/R procedure, SH-SY5Y cells were cultured in glucose-free and serum-free Dulbecco’s modified Eagle medium (DMEM) in an incubator with 100% N_2_ to maintain the oxygen concentration around <0.1% at 37°C for 5 min. Following different periods of OGD (1, 2, and 3 h), ischemic–hypoxic cells were re-incubated in MEM/F12 medium at 37°C under 5% CO_2_ for 24 h.

### 2.3 Determination of cell viability

Cell viability was determined using a CCK-8 cell counting kit (Dojindo, Kyushu, Japan) following the manufacturer’s instructions. The cells were seeded in 96-well plates at 2 × 10^5^ cells/mL and incubated for 24 h. Six wells were used for each treatment and control group. After OGD/R, a mixture of 90 μL of DMEM and 10 μL of CCK‐8 was added to each well. The absorbance of the samples at 450 nm was measured following incubation at 37°C for 2.5 h in the dark.

### 2.4 ROS measurement

Intracellular ROS levels were determined using the 2,7-dichlorofluorescein diacetate (DCFH-DA) fluorescence probe (Beyotime Biotechnology, Shanghai, China). Following treatment, the cells were collected and incubated with 0.1% DCFH-DA in DMEM at 37°C for 20 min. The cells were then washed three times with serum-free DMEM. DCFH fluorescence was recorded in three randomly selected fields. The experiment was repeated three times. The intensity of DCFH fluorescence was assessed using ImageJ Pro Plus 6.0 (Media Cybernetics).

### 2.5 Measurement of total superoxide dismutase (T-SOD) and Manganese- superoxide dismutase (SOD) activities

SOD activity was determined using a SOD assay kit (Nanjing Jiancheng Bioengineering Institute, Nanjing, China) according to the manufacturer’s protocol. The cells were seeded in six-well plates at 1 × 10^6^ cells/well and incubated for 24 h. The cells were then lysed in ice-cold RIPA buffer for 30 min. After centrifugation at 12,000×*g* for 15 min at 4°C, the supernatant was collected. Protein levels were assessed using a BCA protein assay kit (Beyotime Biotechnology). The Cu/Zn-SOD level in the supernatant was determined according to the manufacturer’s instructions. The absorbance of the samples at 550 nm was measured using an ultraviolet spectrophotometer (Youke T2602, Youke Instrument, Shanghai, China).

### 2.6 Detection of mitochondrial membrane potential (MMP)

MMP was examined using a fluorescent probe kit (Beyotime Biotechnology). Briefly, the cells were cultured in six-well plates with JC-1 dye working solution for 20 min at 37°C and then washed twice with JC-1 staining solution. The medium was added to each well. Red (JC-1 aggregates) and green (JC-1 monomer) fluorescence were recorded in three randomly selected fields. Fluorescence intensity was estimated using Image J Pro Plus 6.0.

### 2.7 Observation of mitochondrial structure

The cells were treated with 4% glutaraldehyde at 4°C for 1 h, rinsed twice with pre-cooled phosphate-buffered saline (PBS), and then treated with 1% OsO_4_ for 1 h. The samples were dehydrated with 50% ethanol, 70% ethanol, 80% ethanol, 90% ethanol, 90% ethanol:90% acetone (1:1), 90% acetone, and 100% acetone three times for 10 min per step, and then incubated at 25°C for 1 h. The samples were then embedded in epoxy resin, placed at 35°C for 15 h, then at 45°C for 12 h, and finally at 60°C for 24 h. The cell masses were cut into ultrathin slices and stained. The mitochondrial structure was observed using transmission electron microscopy (Hitachi, Tokyo, Japan), and three different fields were captured.

### 2.8 Determination of apoptosis using Hoechst 33258 staining

Apoptosis was evaluated using a Hoechst 33258 staining kit (Beyotime Biotechnology). The cells were harvested, washed, mixed with 4% (v/v) paraformaldehyde at 25°C for 30 min, further washed, and stained with 500 μL of Hoechst 33258 for 5 min. Fluorescence microscopy (LI-COR, Lincoln, NE, USA) was used to observe nuclear morphology.

### 2.9 Determination of apoptosis using flow cytometry

Apoptosis was measured using flow cytometry with Annexin V-fluorescein isothiocyanate (FITC) and propidium iodide (PI). Early apoptosis of cells was detected by binding Annexin V to phosphatidylserine exposed laterally. PI is a nucleic acid dye that cannot pass through normal cells with intact cell membranes and early apoptotic cells but can pass through the cell membranes of middle and late apoptotic cells and necrotic cells to stain the nucleus. The kit (Beyotime Biotechnology) uses Annexin V combined with PI to distinguish cells at different stages of apoptosis. Flow cytometric analyses were performed using a BD FACSCalibur Flow Cytometer (BD Biosciences; San Jose, CA, USA). Briefly, the cells were seeded in six-well plates at 1 × 10^6^ cells/well. After treatment, the cells were harvested and rinsed twice with PBS. The cells were stained with a 1:1 mixture of V-FITC and PI for 15 min under dark conditions. Thereafter, 400 μL of Annexin V binding buffer was added to each well. Finally, apoptosis was measured using flow cytometry after 1 h.

### 2.10 Western blotting

The cells were lysed in cold RIPA buffer with a 1% protease inhibitor cocktail and phenylmethylsulfonyl fluoride for 30 min. After centrifugation at 13,000×*g* for 15 min at 4°C, the supernatant was collected. Protein expression was measured using a BCA protein assay kit. Identical amounts of protein were loaded into a sodium dodecyl-sulfate-polyacrylamide gel electrophoresis gel and transferred onto a polyvinylidene fluoride membrane. The membrane was blocked in 5% bovine serum albumin for 1 h and then incubated with primary antibodies against phosphor-extracellular-regulated protein kinase 1/2 (p-ERK1/2, 1:2,000), cleaved-caspase 3 (1:500), caspase 3 (1:500), Bax (1:2,000), Bcl-2 (1:500), Drp-1 (1:500), Mfn1 (1:1,000), Opa1 (1:1,000), and Cyto C (1:500) at 4°C overnight. All the primary antibodies were purchased from Cell Signaling Technology. Thereafter, the membrane was incubated with an IRDye800CW-coupled secondary antibody at 25°C for 2 h. Finally, the membranes were visualized using a western blotting detection system with the Li-Cor Odyssey Scanner imaging densitometer (Li-Cor Biosciences, Lincoln, NE, USA), and the assessed bands were quantitated using the ImageJ Pro Plus 6.0. software.

### 2.11 Statistical analysis

Statistical analyses were performed using SPSS software 22.0. Values are presented as mean value ± standard deviation. The Kolmogorov–Smirnov test was used to test for normality and equal variance. All data were processed using a one-way analysis of variance. Results with *p <* 0.05 were considered significant.

## 3 Results

### 3.1 Cellular morphology and cell viability after different durations of oxygen and glucose deprivation (OGD)

**Figure 1:**
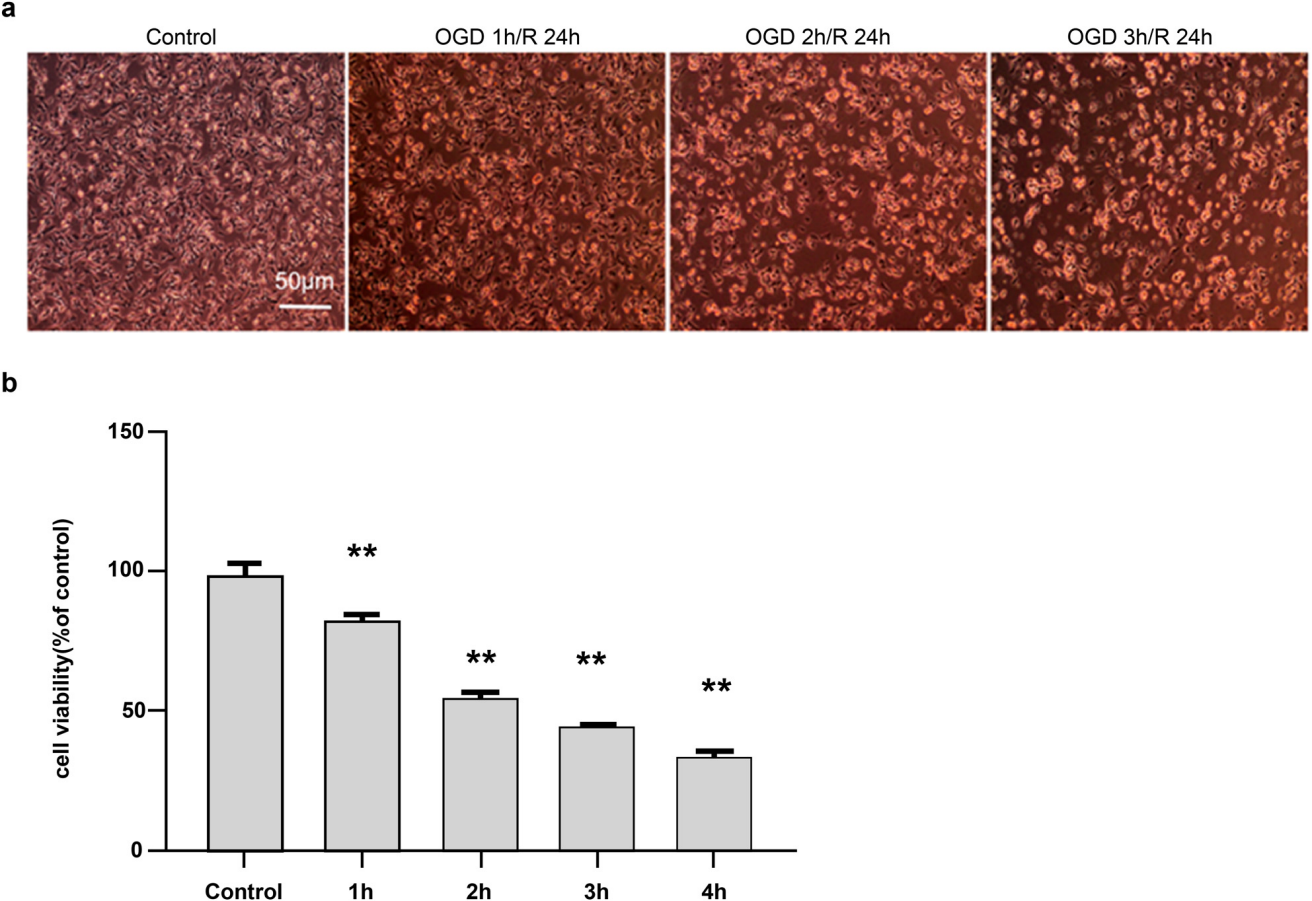
Effect of different durations of oxygen and glucose deprivation (OGD) on cellular morphology and viability. (a) Cellular morphology after different durations of OGD (100×). The morphology of SH-SY5Y cells was observed under an inverted phase-contrast microscope, which revealed a gradual increase in floating cells following OGD. (b) Comparison of cellular viability after different durations of OGD, measured using the CCK-8 method. Scores are presented as mean value ± standard deviation (*n* = 5). **p < 0.01 vs the control group.

Neuroblastoma SH-SY5Y cells were subjected to OGD for 1, 2, and 3 h, followed by reoxygenation for 24 h. Inverted phase-contrast microscopy was used to determine cellular morphology. With prolonged OGD, the number of adherent cells decreased, whereas the number of floating cells increased (Figure 1a). This suggests that OGD markedly affected the morphology of SH-SY5Y cells.

Cell viability was assessed using a CCK-8 kit, according to the manufacturer’s instructions. Compared with that of the control group (100% ± 0.33%), cell viability decreased with prolonged OGD, as follows: 81% ± 0.17% at 1 h, 56% ± 0.25% at 2 h, 35% ± 0.14% at 3 h, and 26% ± 0.10% at 4 h. Considering an optimal cellular density of 50–60% for drug evaluation, cells were subjected to OGD of 2 h in the subsequent experiments (Figure 1b).

### 3.2 ROS levels and p-ERK and ERK expression

**Figure 2:**
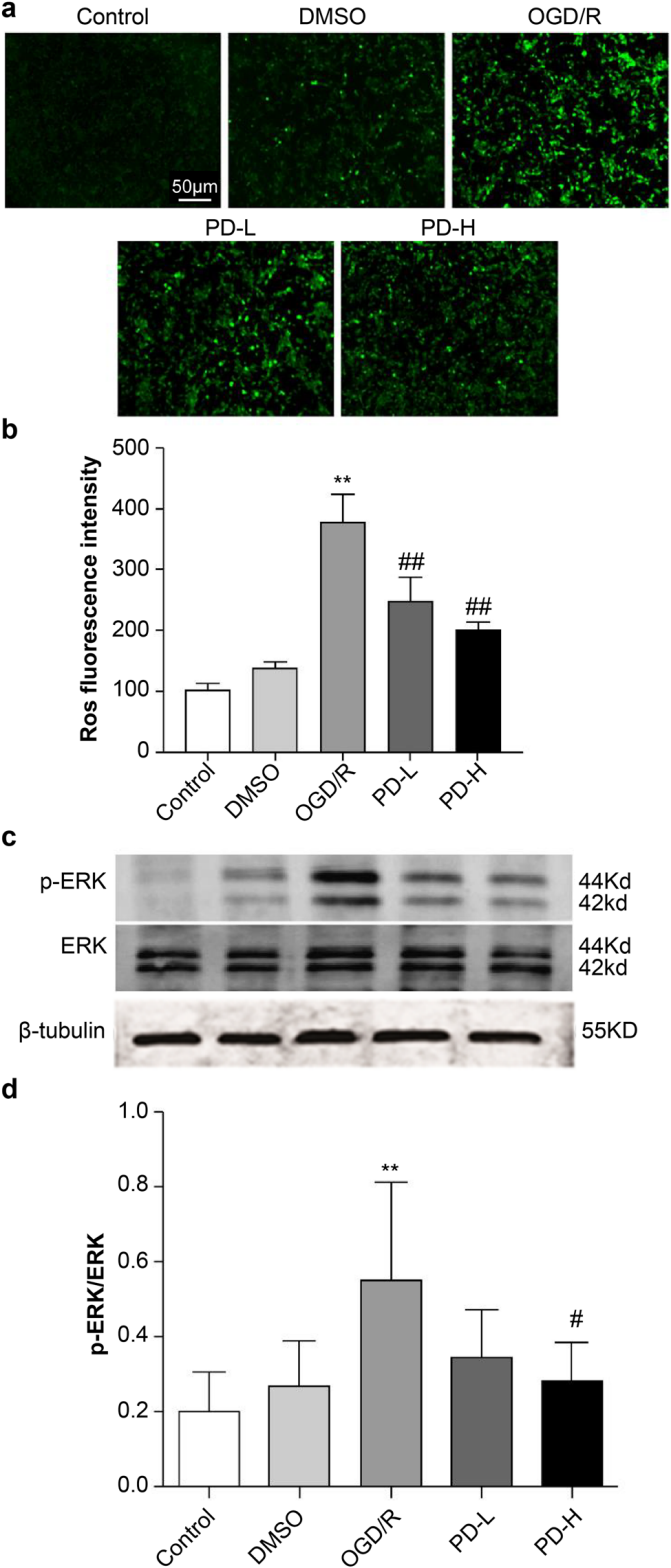
ROS production and p-ERK and ERK expression. (a) ROS fluorescence. (b) ROS fluorescence density. (c) Western blot of p-ERK and ERK. (d) The ratio of p-ERK/ERK. Data are expressed as mean value ± standard deviation (*n* = 3). **p < 0.01 vs the control group; ^##^ *p <* 0.01 vs the oxygen–glucose deprivation/reperfusion (OGD/R) group.

Redox imbalance is a key factor in the development of CIRI. ROS production was determined in SH-SY5Y cells using fluorescence microscopy. The fluorescence intensity was as follows: normal control (control) (101.30 ± 11.48), dimethyl sulfoxide (DMSO) control (DMSO) (137.43 ± 10.89), OGD/R (378.01 ± 45.10), PD low dose (PD-L) (248.69 ± 37.75), and PD high dose (PD-H) (201.99 ± 10.78). OGD/R increased the ROS levels compared with the control (*p <* 0.01). However, treatment with PD reversed these changes (Figure 2a and b). This indicated that oxidative stress is involved in OGD/R. As shown in Figure 2c, the p-ERK and p-ERK/ERK levels increased in the OGD/R group (*p <* 0.01 vs the control group) but decreased in the PD-H group (*p <* 0.05 vs the OGD/R group) (Figure 2c and d).

**Figure 3:**
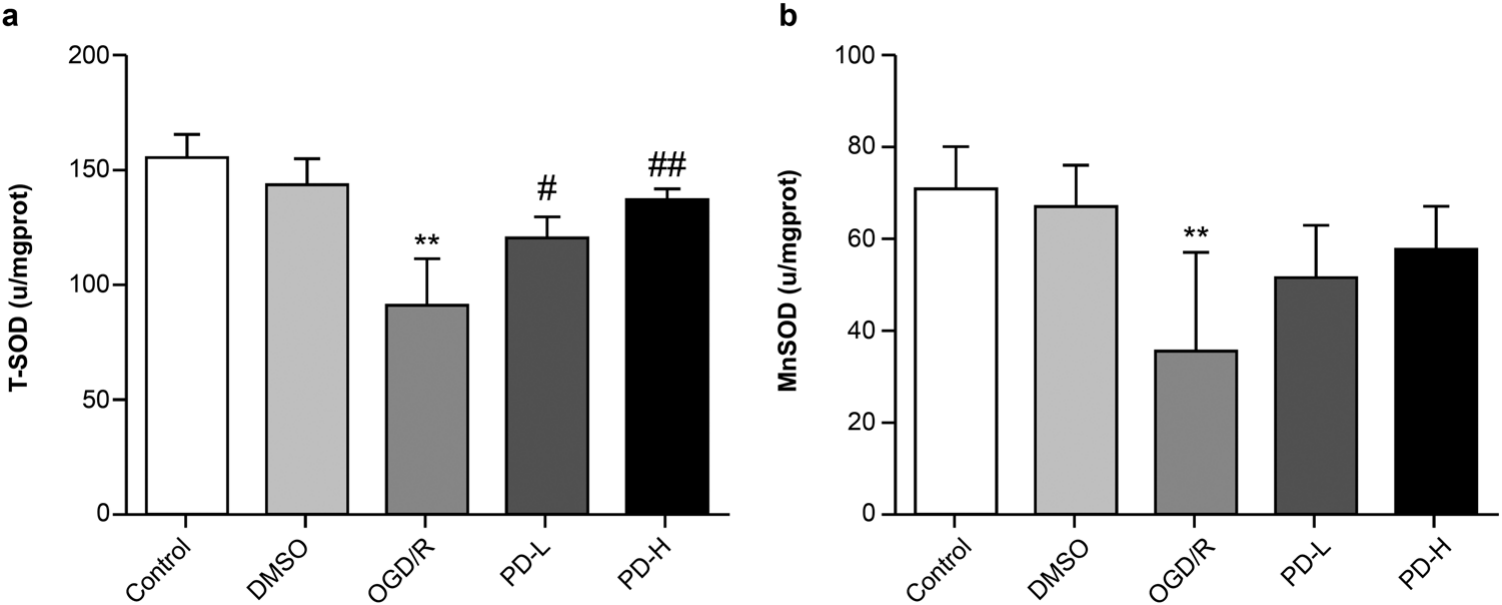
T-SOD and Mn-SOD activities in each group. (a) T-SOD activity. (b) Mn-SOD activity. Data are expressed as mean value ± standard deviation (*n* = 3). **p < 0.01 vs the control group; ^#^ *p <* 0.05 vs the OGD/R group; ^##^ *p <* 0.01 vs the OGD/R group.

### 3.3 Detection of T-SOD and Mn-SOD activities

SOD is a key intracellular antioxidant, and Mn-SOD is an essential mitochondrial antioxidant enzyme. The activities of both T-SOD and Mn-SOD were lower in the OGD/R group than in the control group (*p <* 0.01). The Mn-SOD activity increased in both PD-L and PD-H groups than in the OGD/R group (Figure 3).

### 3.4 Mitochondrial morphology

**Figure 4:**
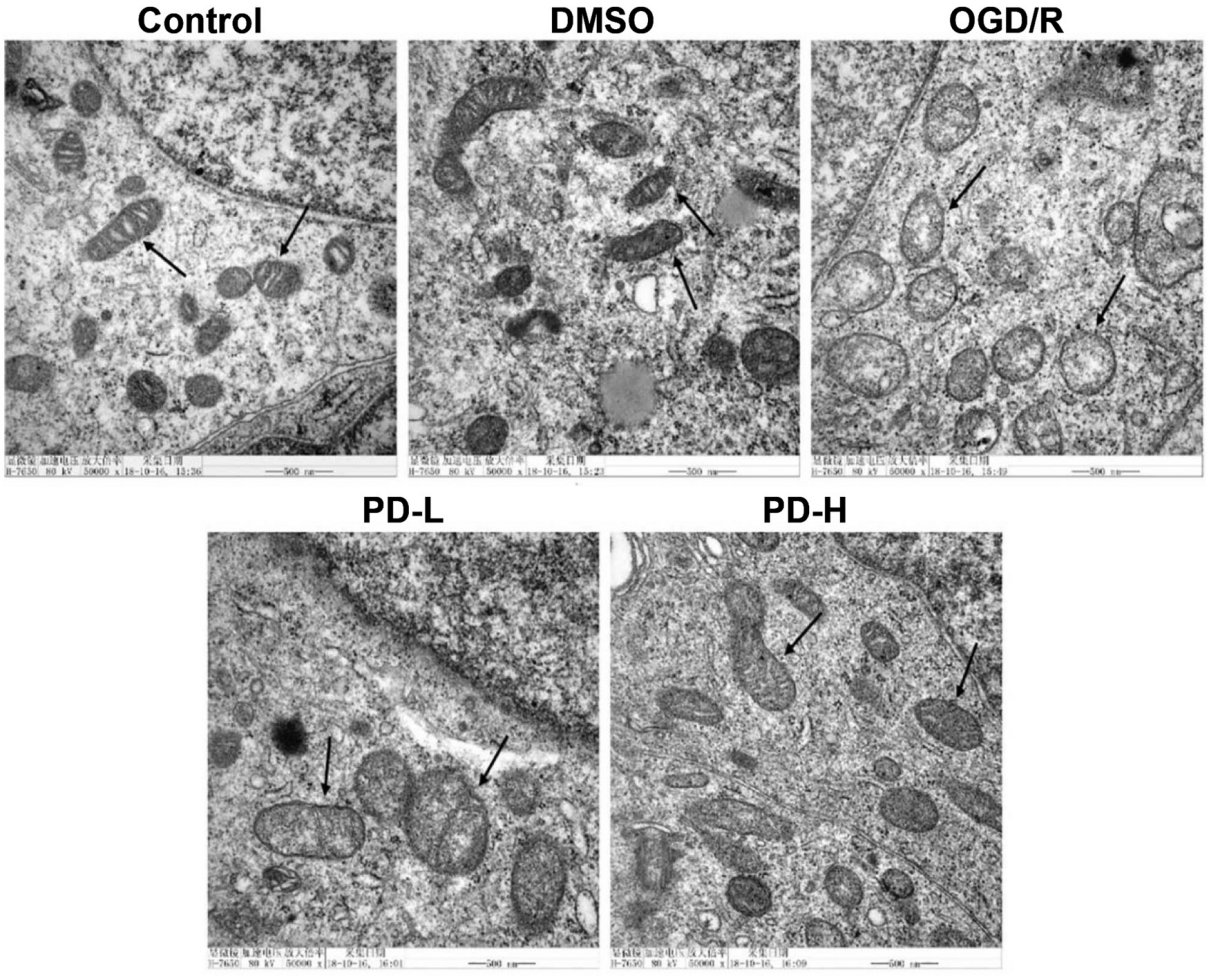
Mitochondrial morphology observed using transmission electron microscopy.

Mitochondrial ultrastructure was observed under an electron microscope. Intact mitochondrial membranes and clear cristae were observed in the control group. Conversely, swollen mitochondria, broken cristae, and vacuolated matrices were observed in the OGD/R group. In the PD-L and PD-H groups, broken cristae and vacuolization improved compared with those in the OGD/R group (Figure 4).

Mitochondrial images were obtained under an electron microscope at 50,000×. Arrows indicate a single mitochondrion.

### 3.5 MMP

**Figure 5:**
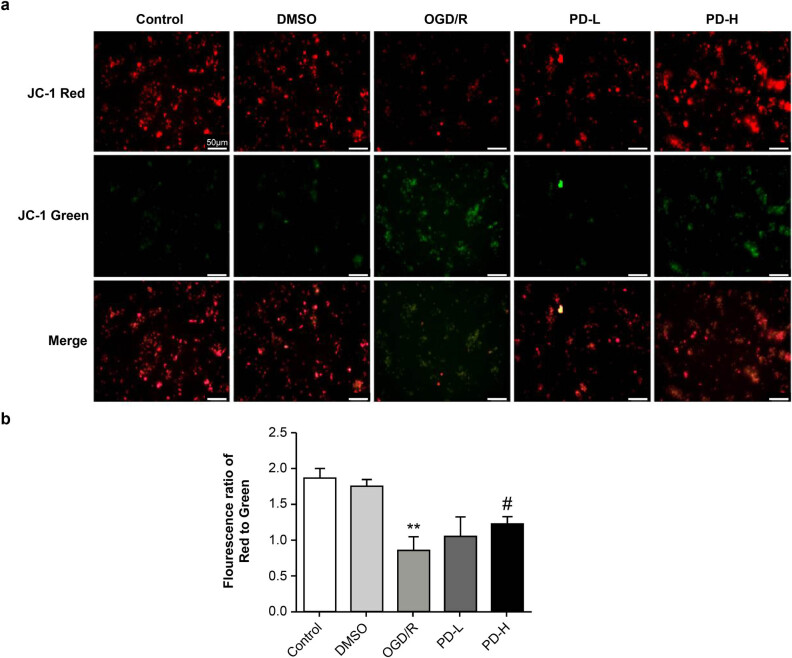
Mitochondrial membrane potential. (a) Determination of membrane potential using fluorescence microscopy. Images were acquired at 100×. (b) The relative amount of red/green fluorescence was calculated using ImageJ software. Data are presented as mean value ± standard deviation (*n* = 3). **p < 0.01 vs the control group; ^#^ *p <* 0.5 vs the OGD/R group.

A double immune marker was used to evaluate MMP. Red staining indicated a higher potential, whereas green staining indicated a lower potential, denoting early-stage apoptosis. The red and red/green ratios of the OGD/R group decreased compared with those in the control group (*p <* 0.01), indicating lower MMP levels. A significant enhancement was observed in the MMP in the PD-H group compared with that in the OGD/R group (*p <* 0.05) (Figure 5).

### 3.6 Measurement of optic atrophy 1 protein (Opa1), mitofusin 1 (Mfn1), and dynamin-related protein 1 (Drp1) levels

**Figure 6:**
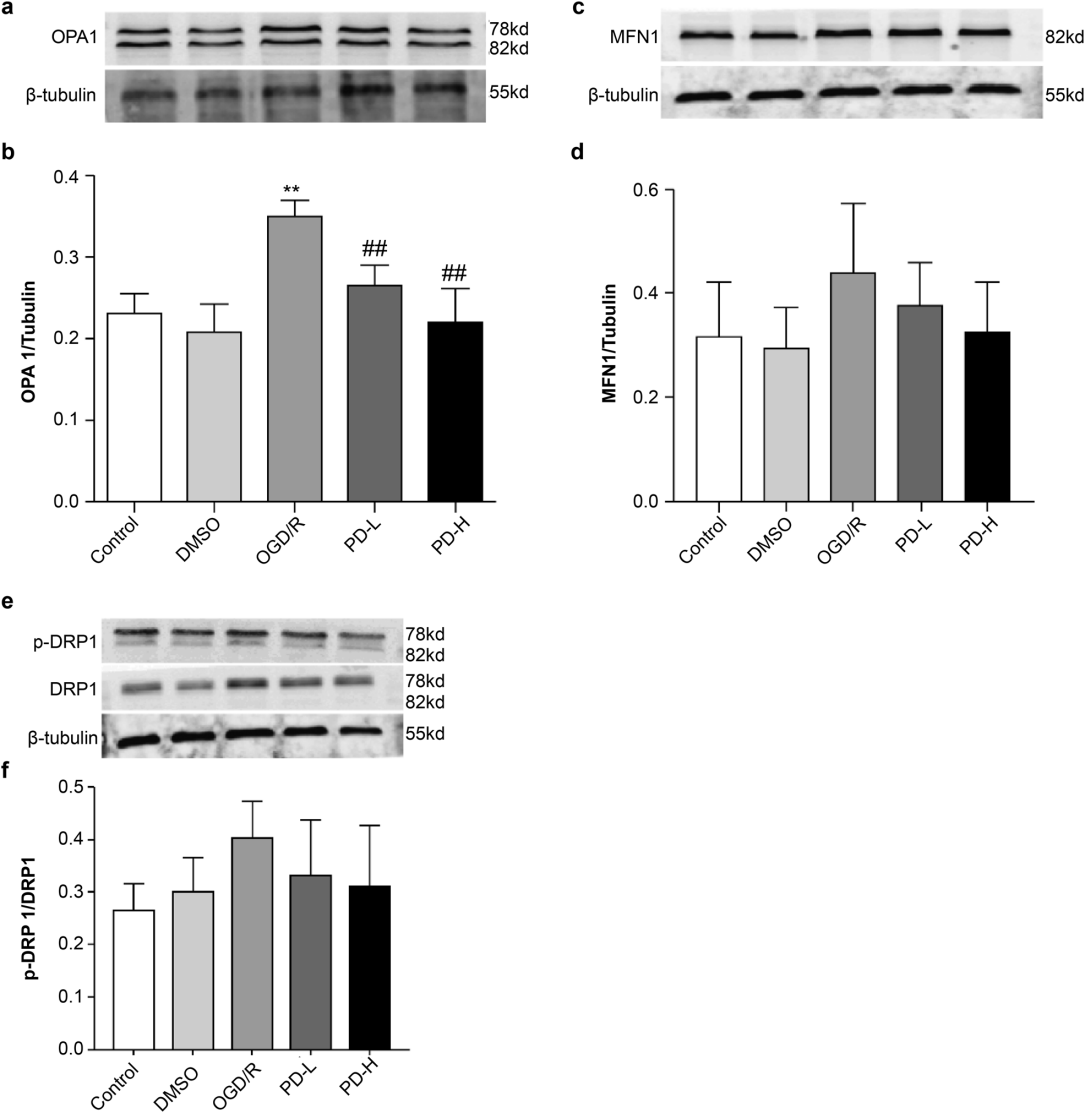
Western blotting of the mitochondrial proteins Mfn1, Opa1, and Drp1. (a) Western blotting of Opa1. (b) Quantification of Opa1 band intensity. (c) Western blotting of Mfn1. (d) Quantification of Mfn1 expression. (e) Western blotting of the mitochondrial fission proteins p-Drp1 and Drp1. (f) Ratio of p-Drp1/Drp1 expression. Overall data are expressed as mean value ± standard deviation (*n* = 3). **p < 0.01 vs the control group; ^##^ *p <* 0.01 vs the OGD/R group.

Western blotting was performed to analyze Opa1, Mfn1, and Drp1 levels, which mediate mitochondrial fusion and fission. OGD/R increased Opa1 expression compared with the control (*p <* 0.01). Opa1 expression decreased considerably in the PD-L and PD-H groups compared with that in the OGD/R group (*p <* 0.01) (Figure 6a and b). Compared with the control, OGD/R also significantly increased the expression of Mfn1. Both PD-L and PD-H groups showed a downward trend in Mfn1 expression (Figure 6c and d). Compared with the control, OGD/R increased p-Drp1/Drp1, whereas PD-L and PD-H had the opposite effect (Figure 6e and f).

### 3.7 Hoechst staining and flow cytometry analysis of cell apoptosis

**Figure 7:**
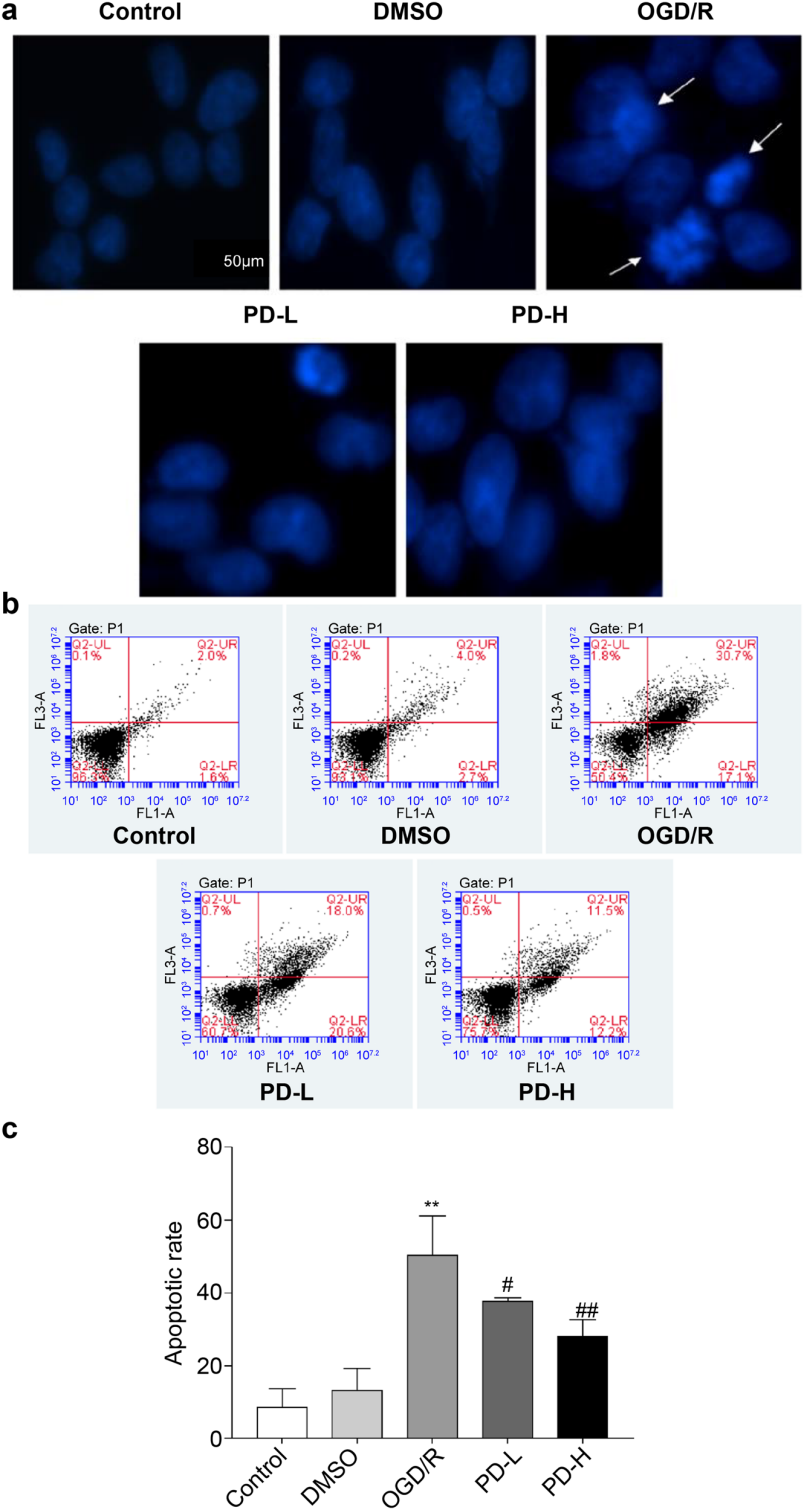
Observation of cell apoptosis. (a) Cell apoptosis observed using Hoechst staining. Images were acquired at 400×. Arrows indicate damaged nuclei. (b and c) The rate of apoptosis was determined using flow cytometry. Data are presented as mean value ± standard deviation (*n* = 3). **p < 0.01 vs the control group; ^#^ *p <* 0.05 vs the OGD/R group; ^##^ *p <* 0.01 vs the OGD/R group.

No significant differences in nuclear morphology were observed in the DMSO group compared with that in the control group; however, in the OGD/R group, the staining pattern was dispersed and deep, indicating that the nuclei were disrupted. Compared with OGD/R, PD-L and PD-H significantly improved nuclear morphology (Figure 7a).

Flow cytometry was performed to investigate the effect of PD on apoptosis after OGD/R. The rates of apoptosis were as follows: control group, 8.5% ± 5.2%; DMSO group, 13.4% ± 5.8%; OGD/R group, 50.4% ± 10.7%; PD-L group, 37.6% ± 1.1%; and PD-H group, 27.9% ± 4.6%. Both PD-L and PD-H decreased the rate of apoptosis compared with OGD/R (*p <* 0.05 and *p <* 0.01, respectively) (Figure 7c).

### 3.8 Cytochrome C (Cyto C), caspase 3, cleaved-caspase 3, Bax, and Bcl-2 expression

**Figure 8:**
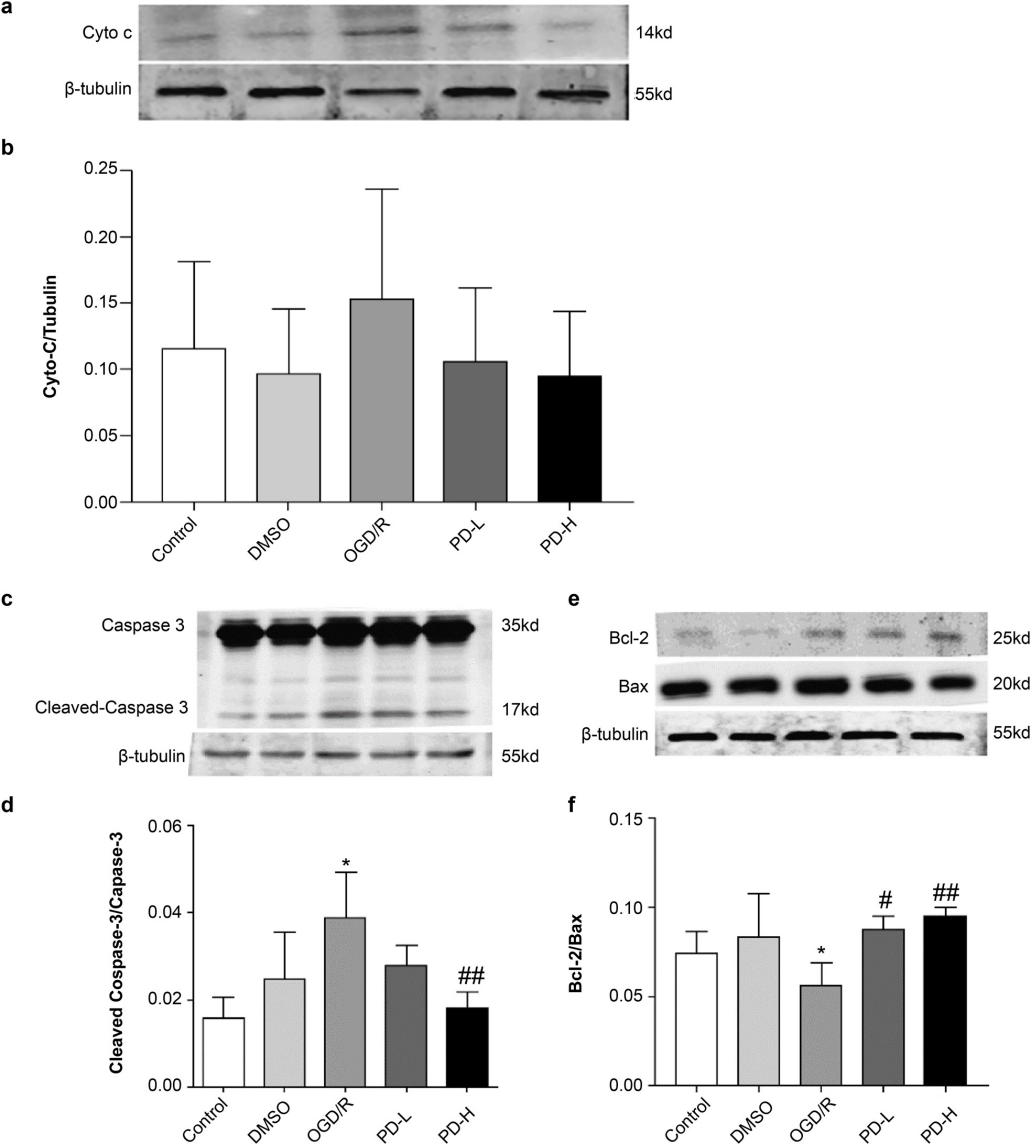
Western blotting of mitochondrial Cyto C, cleaved-caspase 3, caspase 3, Bcl-2, and Bax. (a) Western blotting of Cyto C. (b) Quantification of Cyto C band intensities. (c) Western blotting of cleaved-caspase 3 and caspase 3. (d) Ratio of cleaved-caspase 3/caspase 3 band intensities. (e) Western blotting of Bcl-2 and Bax. (f) Bcl-2/Bax quantification. Values are presented as mean value ± standard deviation (*n* = 3). *p < 0.05 vs the control group; ^#^ *p <* 0.05 vs the OGD/R group; ^##^ *p <* 0.01 vs the OGD/R group.

The release of Cyto C from the mitochondria to the cytosol is a critical marker of mitochondrial membrane permeability and mitochondria-pathway apoptosis. We found that OGD/R increased Cyto C expression, whereas this was inhibited by PD (Figure 8). The levels of cleaved-caspase-3 and Bcl-2/Bax in the OGD/R group were considerably higher than those in the control group (*p <* 0.05). PD-H lowered the cleaved-caspase-3 level compared with OGD/R (*p <* 0.01), and both PD-L and PD-H increased the Bcl-2/Bax level compared with OGD/R (*p <* 0.05 and *p <* 0.01, respectively) (Figure 8).

## 4 Discussion

This study proved that the ERK inhibitor PD reduced ROS production, maintained mitochondrial morphology and function, and alleviated the mitochondria-dependent apoptosis in SH-SY5Y cells caused by OGD/R. Brain injury following CA/CPR remains a major concern worldwide. The findings of our previous study indicated that PD plays a brain-protection role in a rat model of CA/CPR [12]. To explore the effect of PD *in vitro*, we established an OGD/R model and found that PD alleviated the ROS production following OGD/R injury, mitochondrial injury, and apoptosis in SH-SY5Y cells.

Oxidative stress is an important pathological factor that increases with the changes in mitochondrial function during CIRI [17]. Oxygen deficiency inactivates the mitochondrial proteins of the electron transfer chain, resulting in proton leakage. Following reperfusion, protons react with O_2_, resulting in the overproduction of ROS [18,19]. Inactivation or overconsumption of endogenous antioxidative enzymes promotes ischemia-reperfusion injury [2]. Therefore, in this study, PD prevented OGD/R-induced ROS production and Mn-SOD inactivation by protecting the mitochondria. This is consistent with our previous findings: PD plays an antioxidant role via a decrease in ROS production and an increase in the SOD level in a rat model of CA/CPR [20].

Mitochondrial function plays a vital role in cellular fate. Mitochondria-dependent apoptosis is common during ischemia-reperfusion [21,22]. MMP depends on membrane ion transporters, which are sensitive to ischemia. A decrease in MMP, an important index of mitochondrial dysfunction and a hallmark of early apoptosis [23], leads to the transformation of apoptosis-promoting proteins, including Cyto C, further activating apoptotic Bax and cleaved-caspase-3 [24,25]. In this study, PD improved the mitochondrial ultrastructure, increased MMP, reduced Cyto C release, and downregulated Bax and cleaved-caspase-3 expression.

Mitochondria are highly dynamic, exhibiting fusion and fission [26,27]. Mitochondrial fusion allows the exchange and connection of mitochondrial contents to form normal mitochondria, regulated by Mfn1, Mfn2, and Opa1. Mitochondrial fission, which is a requisite for cell growth and division, results in sufficient numbers of mitochondria, eliminates impaired mitochondria, and is mainly regulated by Drp1. Experiments have shown that mitochondrial fusion and fission are involved in regulating apoptosis. Silencing Mfn, Opa1, and Drp1 upregulates or downregulates the sensitivity of mitochondria to apoptotic stimuli [28,29,30]. Several studies have reported that mitochondrial dynamics are imbalanced following CIRI [31,32]. Modulation of the mitochondrial fusion/fission balance positively affects brain injury. Pretreating a rat model of CIRI with a mitochondrial fusion promoter increases the expression of Mfn1 and reduces the dysfunction and apoptosis of brain mitochondria [32]. Resveratrol acts on the AMPK-Mfn1 pathway to exert antioxidative and antiapoptotic effects in N2a cells subjected to hypoxia-reoxygenation injury. PD upregulates Mfn2, downregulates P-Drp1, and positively labels P-Drp1/TUNEL cells in the cerebral cortex of CA/CPR rats [11,12]. A Drp1 inhibitor (Mdivi-1) or siRNA has been shown to inhibit the mitochondria-dependent apoptotic pathway following cerebral ischemia or CA [30,33]. Drp1 knockdown protects mouse N2a cells against mitochondrial damage and apoptosis during OGD [34]. In this study, SH-SY5Y cells subjected to OGD/R presented increased levels of Opa1, Drp1, and Mfn1, which were reversed by PD. This likely reflected the condition at 24 h of reperfusion due to fluctuating mitochondrial dynamics during ischemia-reperfusion injury. Liu et al. [35] revealed that the expression of Opa1 and Drp1 increased in mouse ischemic penumbra 90 min after transient middle cerebral artery occlusion, followed by a fastigium at 2 days, whereas the expression of P-Drp1 gradually increased, peaking at 14 days. Experimental results may vary among different models depending on the duration and severity of cerebral ischemia.

Although an emerging effect was observed in the total expression of Opa1, Mfn1, and Drp1, the number of western blot repeats was low, which is a limitation of the current study. Thus, dynamic variations in Opa1, Mfn1, and Drp1 levels require observation from multiple time points; however, this report only observed the expression of these proteins during OGD of 2 h.

## 5 Conclusion

Collectively, our results indicate that the ERK inhibitor PD protected SH-SY5Y cells from OGD/R damage by reducing ROS production, maintaining mitochondrial morphology and function, and alleviating mitochondria-dependent apoptosis. The present findings, together with those of our previous studies in CA/CPR rats, imply that the ERK inhibitor and mitochondrial may be the potential therapeutic target in CIRI.
